# Effects of Divacancy and Extended Line Defects on the Thermal Transport Properties of Graphene Nanoribbons

**DOI:** 10.3390/nano9111609

**Published:** 2019-11-13

**Authors:** Min Luo, Bo-Lin Li, Dengfeng Li

**Affiliations:** 1 Chongqing Key Laboratory of Extraordinary Bond Engineering and Advanced Materials Technology (EBEAM), Yangtze Normal University, Chongqing 408100, China; minluo@yznu.edu.cn; 2 School of Science, Chongqing University of Posts and Telecommunications, Chongqing 400065, China

**Keywords:** thermal transport, graphene, divacancy, extended line defect, nonequilibrium Green’s function

## Abstract

The effects of divacancy, including isolated defects and extended line defects (ELD), on the thermal transport properties of graphene nanoribbons (GNRs) are investigated using the Nonequilibrium Green’s function method. Different divacancy defects can effectively tune the thermal transport of GNRs and the thermal conductance is significantly reduced. The phonon scattering of a single divacancy is mostly at high frequencies while the phonon scattering at low frequencies is also strong for randomly distributed multiple divacancies. The collective effect of impurity scattering and boundary scattering is discussed, which makes the defect scattering vary with the boundary condition. The effect on thermal transport properties of a divacancy is also shown to be closely related to the cross section of the defect, the internal structure and the bonding strength inside the defect. Both low frequency and high frequency phonons are scattered by 48, d5d7 and t5t7 ELD. However, the 585 ELD has almost no influence on phonon scattering at low frequency region, resulting in the thermal conductance of GNRs with 585 ELD being 50% higher than that of randomly distributed 585 defects. All these results are valuable for the design and manufacture of graphene nanodevices.

## 1. Introduction

Due to its excellent mechanical, electrical and thermal properties, graphene has broad application prospects in many fields [1,2,3,4,5]. During the growth of graphene, although the C-C bond is strong, there are still some structural defects [6], among which divacancies are more energy-stable and common [7,8]. The formation energy of a divacancy in graphene is much less than two mono-vacancies [9,10]. Thus, divacancy is easier to form than mono-vacancy, and two relatively nearby mono-vacancies have the tendency to approach and form a divacancy [10,11]. Therefore, it is particularly important to study the effect of divacancies on the performance of graphene.

It has been found that divacancies can affect the mechanical [12,13,14,15], electrical [16,17,18], magnetic [19,20] and chemical [21,22] properties of graphene. Some of these effects can provide some useful performance qualities. For example, it can be used to produce field effect transistor [23,24] or negative differential resistance devices [25], to filter the electronic or phononic valleys [26,27], and to introduce [20] or modulate [28,29] magnetic characteristics. Divacancy can adsorb some gas molecules [21,22], which indicates that it can be used to produce gas sensors [30]. Therefore, in order to take advantage and employ divacancies, we will intentionally introduce them into graphene.

Heat dissipation is the most important factor affecting and limiting the performance of modern electronic devices [31,32,33,34,35,36]. Thus, how divacancies affect heat transport performance of graphene and graphene nanodevices is an important question. Zhang et al. found that 8.25% of mono-vacancy can reduce thermal conductivity by 3 orders of magnitude [37]. Jiang et al. found that a mono-vacancy defect situated at the center of the nanoribbon will have a greater effect on the thermal conductance than at the edge [38]. Haskins et al. found that 0.1% of the vacancy defect caused a significant reduction in thermal conductivity [39]. So far, regarding the effect of divacancy on the thermal transport of graphene, only the 585 (two pentagons and one Octagon) divacancy was studied [39,40,41]. It was found that 10% of divacancy defects reduce the thermal conductivity of graphene by 80%, and the decrease of the zigzag graphene nanoribbon is greater than that of the armchair type in the case of high defect concentration [41]. However, experimental results show that both 585, t5t7 (three pentagons and three heptagons) and f5f7 (four pentagons, four heptagons and one hexagon) divacancies are stable and common in graphene [7,42,43], and they can propagate in the structure [44,45,46] or transform each other [42,43,47]. Therefore, it is necessary to systematically study the effect of divacancies with different structures on the thermal transport properties of graphene.

In addition, multiple divacancies in a regular arrangement can form chain-like line defects [48,49], known as extended line defects (ELDs). Four types of extended line defects (585, d5d7 and t5t7 defects for armchair GNRs, 48 defects for zigzag GNRs) were mainly observed in experiments [8,50], and the formation of chain structure could be controlled precisely [51]. Extended line defects tend to provide graphene with another boundary [51,52], which can be used to control magnetic characteristics of GNR [28,29,53,54] or make graphene behave as one-dimensional metallic wires [50]. The influence of the 585, d5d7 and t5t7 ELD on the thermal transport properties of the armchair graphene nanoribbon was reported in Ref. [55], and it was found that the thermal conductance could be effectively regulated by controlling the orientation or changing the length of the embedded long chain. The decrease of thermal conductance is caused by the change of phonon dispersion and the scattering of defects. However, the influence of 48 extended line defects on the thermal transport properties of the zigzag graphene nanoribbon has not been reported. We can compare the influence of extended line defects on the thermal transport properties of graphene nanoribbons with different boundaries, and analyze the difference between multiple random divacancies and extended line defects, which are useful for the thermal regulation of electronic devices based on graphene nanoribbons.

## 2. Methods

The nonequilibrium Green’s function (NEGF) method is used to study the elastic phonon transport properties of graphene nanoribbons herein, which is appropriate to study the thermal transport properties of nanoscale junctions [56]. It is a successful method to describe quantum thermal transport in nanosystems, such as nanowires [57,58], nanotubes [59,60], nanoribbons [61,62], the nanostructures with isotope, defects [63,64,65,66] or strain [67]. In this method, the thermal conductance is calculated through the Landauer Formula, [60,68,69]

(1) $$\sigma =\frac{\hbar^{2}}{2\pi k_{B}T^{2}}\int_{0}^{\infty }d\omega \frac{\omega^{2}e^{\hbar \omega /\left(k_{B}T\right)}}{{\left(e^{\hbar \omega /(k_{B}T)}-1\right)}^{2}}T\left(\omega \right).$$

where the phonon transmission coefficient of a junction is defined by the retarded/advanced Green’s function $G^{r/a}$ of center part and the broadening function $\Gamma_{L/R}$ from the coupling to left/right heat bath:

(2) $$T\left(\omega \right)=\mathrm{Tr}\left[G^{r}\left(\omega \right)\;\Gamma_{L}\left(\omega \right)\;G^{a}\left(\omega \right)\;\Gamma_{R}\left(\omega \right)\right].$$

The force constant of device is calculated by General Utility Lattice Program (GULP) [70] using the classical Brenner potential [71], which is a good description of phonons in graphene nanosystems. The structure of GNR is fully relaxed until the forces on the atoms are below 0.02 eV/Å.

## 3. Results and Discussion

### 3.1. Single Divacancy

We choose 15-ZGNR (graphene nanoribbon with 15 zigzag carbon chains in width direction) and 26-AGNR (graphene nanoribbon with 26 dimer carbon chains in width direction) as the research objects. The nanoribbon width is an influencing factor of tuning the thermal conductance of GNRs [61,72,73], so we choose the comparable width of 3.20 nm for different edges. The channel length in the center region is chosen as 3.7–3.8 nm for devices with only a single divacancy. When the nanoscale channel length is smaller than phonon mean free path, phonons will transport ballistically or quasi-ballistically, and the thermal conductance will remain almost constant regardless of the channel length. Ballistic transport could even be effective for microscale channels [74,75,76,77]. In this paper, we only discuss the ballistic thermal transport and we have also checked that this increase in length has not influenced the thermal conductance of single divacancy.

**Figure 1:**
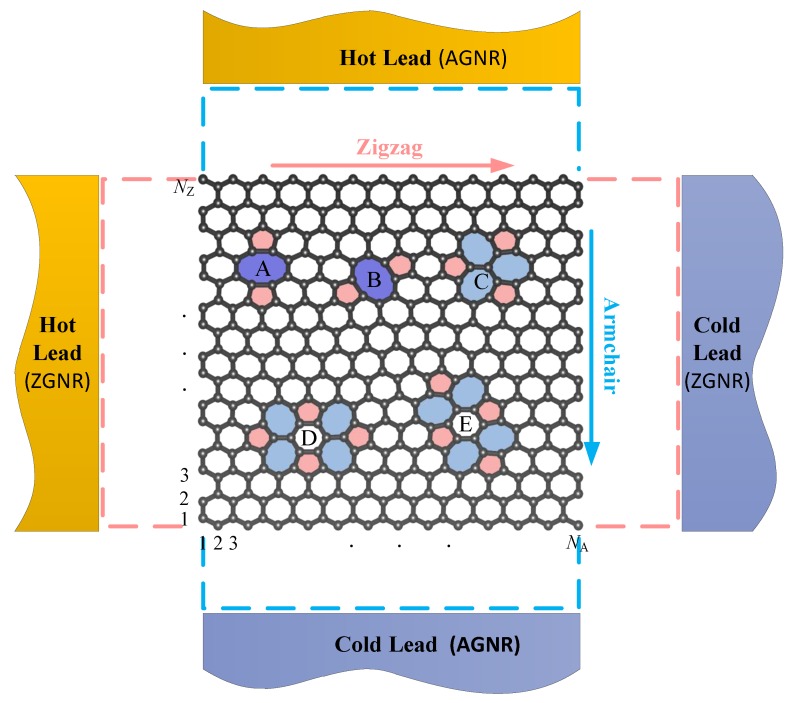
The structure diagrams calculated herein. Zigzag (armchair) nanoribbon, is described by the central structure, the left and right (upper and lower) dotted frames and leads, and the upper and lower (left and right) dashed boxes and leads are null. Notices that, this figure just demonstrates the structure and direction of divacancies which are calculated here, not the exact position of the device. We will set the position almost at the same distance from the two leads in the transport direction, and at the center or the edge of the width direction. Convenient for comparing different boundaries, the 15-ZGNR and 26-AGNR, with width of approximately 3.20 nm, are chose as the research objects.

Three different configurations of divacancies: 585, t5t7 (555777) and f5f7 (5555-6-7777) are taken into account, including diverse directions of the 585 and f5f7 divacancy. The 585 divacancy (Figure 1, A and B)) contains a pair of pentagons and one octagon, the t5t7 divacancy (Figure 1 C) contains three pentagons and three heptagons, the f5f7 divacancy (Figure 1, D and E) contains four pentagons and four heptagons. It’s found that there are five kinds of divacancy configurations (shown in Figure 1) in the GNRs when considering the symmetry of transverse mirror and left-right lead inversion, where B and E are the structures obtained by rotating A and D at a certain angle respectively.

**Figure 2:**
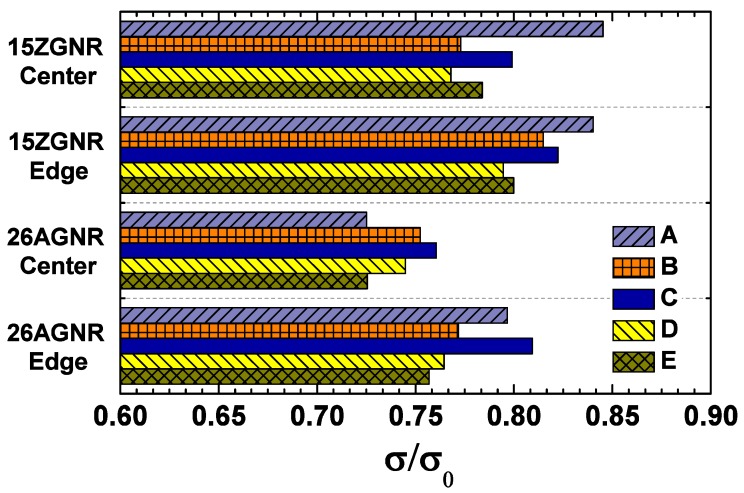
Ratio of the thermal conductance of graphene nanoribbon containing a single divacancy σ to the thermal conductance of pristine graphene nanoribbon $\sigma_{0}$.

The thermal conductances of pristine 15-ZGNR and 26-AGNR are investigated by the nonequilibrium Green’s function approach firstly, which are similar to the original results [61]. The thermal conductance of the ZGNR is higher than that of the AGNR, which indicates the existence of strong anisotropy of thermal conductance. As showing in Table 1 and Figure 2, we have obtained the thermal conductances of the GNRs with different types of single divacancy. The thermal conductances have been greatly reduced due to the scattering of defects. The largest decline is model A or E in the middle zone of 26-AGNR, which is decreased to approximately 73% of the pristine GNR. In general, we can find that the thermal conductance of AGNR is more affected by divacancy defects than that of ZGNR. This phenomenon is similar to the previous observation in graphane nanoribbons [78]. That is the variance in boundary condition affects the strength of the impurity scattering. It may be understood as the collective effect of impurity scattering and boundary scattering. The anisotropic thermal conduction in pristine graphene nanoribbons originated from the strength of boundary scattering, that is, the scattering of armchair boundary is stronger than that of the zigzag [61]. When an impurity is introduced in the structure, the impurity could change the transport direction of some phonons, and make more phonons meet the edge of GNRs, which means more boundary scattering. Armchair boundary scatter stronger than zigzag, then the thermal conductance decreases faster than zigzag.

The effect of divacancy on the thermal transport of GNR is closely related to the location of defects. It is found that the thermal conductance of GNR with a divacancy at the ribbon edge is greater than that at the center, except for the case of model A in the 15-ZGNR, which indicates that the influence of the divacancy at the edge on the thermal transport properties of the GNR is less than that in the middle of ribbon width. Although the thermal conductance of the 15-ZGNR with a type-A divacancy at the edge is almost the same as that in the center region, the thermal conductance of the 26-AGNR with a type-A divacancy at the boundary is much larger than that in the center region. In addition, we have further studied the influence of divacancies in different directions. For the 585 divacancy, the change in thermal conductance strongly depends on its direction; while the direction of the f5f7 divacancy has little influence on the thermal conductance. Whether the 585 divacancy is in the center or at the edge of the 15-ZGNR, when the divacancy is perpendicular to the heat flow, the effect on thermal conductance is much smaller than when the divacancy is at an angle of 30${}^{\circ }$ to the heat flow.

**Figure 3:**
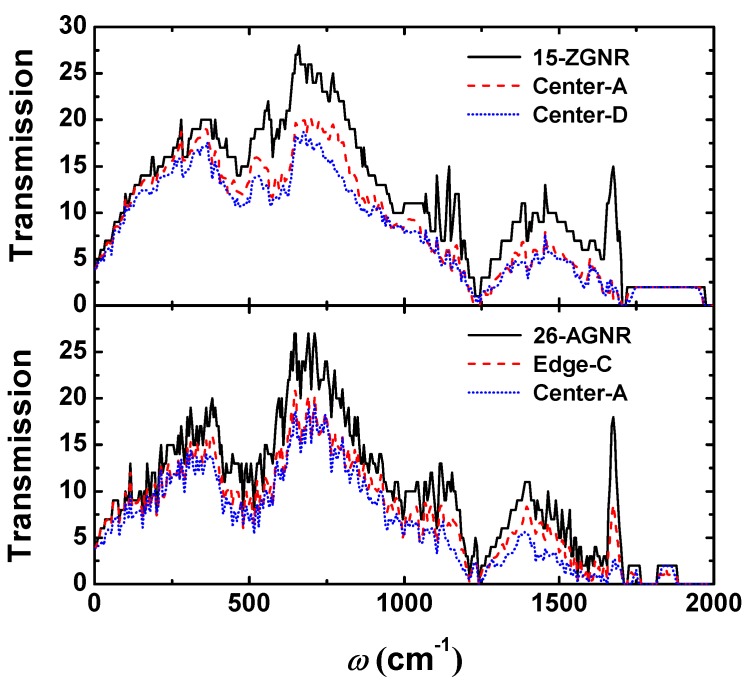
Transmission of graphene nanoribbon containing a single divacancy with minimal (15-ZGNR: Center-A, 26-AGNR: Edge-C) and maximum (15-ZGNR: Center-D, 26-AGNR: Center-A) influence on thermal conductance.

We can explain the downward trend of the thermal conductance by phonon transmission. The phonon transmission of the 15-ZGNR and the 26-AGNR is shown in Figure 3. We can notice that the influence of the divacancy on the phonon transmission is mainly in the high frequency region and has little effect on the low frequency phonons, which indicates that scattering of high frequency phonons caused by divacancy is strong. For ZGNRs and AGNRs, both of the differences between the greatest influence and least influence on the phonon scattering are mainly concentrated in the high frequency region.

From the previous results and discussions, the influence of a single divacancy on thermal conductance can be summarized into three aspects: (1) The difference of boundary will cause significant difference in thermal conductance; (2) The type difference of divacancy (including direction) will produce different impact; (3) the same divacancy structure, placed in different positions (center or edge), the impact on the thermal conductance will be different. The first tendency has been explained by the collective effect of impurity scattering and boundary scattering in previous text. Then, how to understand the latter two conclusions? In particular, the influence of different types of double vacancies seems complicated, that is, the ranking of the influence of each divacancy is fickle. Therefore, it is crucial whether you can find a relatively simple method to understand those phenomenons.

**Figure 4:**
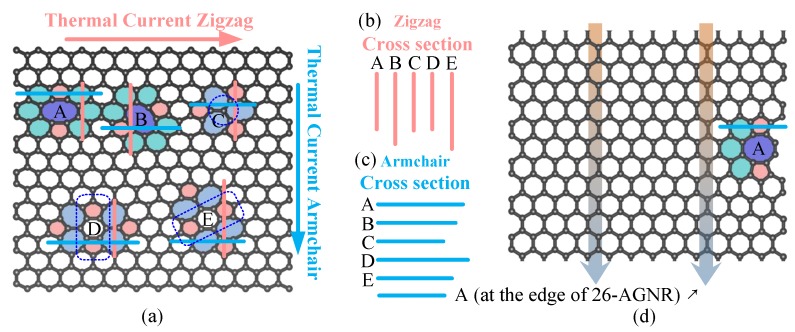
The cross section in front of the thermal current of various divacancies. (**a**) The structure of divacancies in the center. (**b**,**c**) The aligned cross section lines translated from subfigure (**a**). (**d**) The structure of type A 585 divacancy located at the edge of 26-AGNR.

After repeated and careful analysis, we found that it may be understood along the following approach. When phonons move in a system with scatterers (defects or impurities), if they encounter with a scatterer, some phonons can move forward through the scatterer, and the others will be scattered, which will change direction or even move backward. Scattering is the intrinsic mechanism of how defects affect the properties of thermal transport. So what kind of defects will have a stronger scattering ability? First of all, the size of the defect will affect its ability to scatter. More precisely, it is the size of the cross section of the defect, or be called as the effective size, in the view along the direction of the phonon motion. For two-dimensional materials, the cross section of a defect can be measured by the width of the defect in the direction perpendicular to the transport path, seeing Figure 4a. Obviously, if the cross section (width) is larger, more phonons will collide with the defects; and if the cross section is smaller, more phonons will smoothly pass through the side without being affected by the defect. Thus, if ignoring the specific structural inside defects, only from the view point of the cross-sectional size, it can be expected that the larger cross section of a defect, the more phonons collide with the defect, so there may be more phonons scattered, resulting in a stronger effect on thermal conductance. Secondly, the cross-sectional size of the defect only describes how many phonons will collide with the defect, then it is necessary to do a more specific analysis to answer the question of whether the phonon penetrates the defect or will be scattered away. Specifically, it is necessary to analyse the structure inside the defect and the strength of chemical bonds. In general, the smaller the damage to the original structure, or the stronger the internal bonds, the better the phonons are able to penetrate and the less they are scattered; otherwise, they are less able to penetrate and more likely to be scattered.

Using this analytical method of combining the cross-sectional size and the internal structure of defects, we can analyze how the various divacancies affect the thermal conductance and explain why the divacancy at the edge has less influence on the thermal conductance than that in the center. To measure the size of cross section, we depict the cross-section (effective width) of various defects in the direction perpendicular to the transport direction, seeing the lines in Figure 4a. These lines are translated and aligned to facilitate subsequent comparative analysis. What needs to be specifically stated here is, unlike in the drawing in Figure 1, we also classify the hexagons around the 585 divacancies into their size. The reason for this is that these hexagons are severely distorted due to the introduction of 585 divacancies. If only the two pentagons and one Octagon are taken into account, the influence of a 585 divacancy will be seriously underestimated. The hexagons around other kinds of divacancies (t5t7 and f5f7)are not included in their size due to the deformation of these hexagons is much weaker than that of 585 divacancies. Now we will illustrate how to explain the results in Figure 2 through this combined method of the cross-sectional size and the internal structure:

**Example** **1.** *Describe and explain the thermal conductance data when divacancies are located at the center of the 15-ZGNR. The cross sections of the type A, C and D divacancies are similar, and they are the smallest. Fewer phonons will collide with those defects, and their scattering may be weaker, and their thermal conductance may be larger. Comparing A, C and D, we can judge which defect allows the phonon to penetrate more easily by examining their internal structure. For A, the zigzag atomic chains remain connected and have not been interrupted; For C, the zigzag atomic chains are broken, and the central three bonds (marked by the dashed circle in Figure 4a) are relatively weak. Thus, more phonons penetrate through A and less scattered by A, then the thermal conductance of A is larger than C. For D, the central hexagon is connected to the top and bottom atoms with two bonds, and only with one bond to the left and right side atoms, respectively. This indicates that the strength in the part marked by dashed line in Figure 4a is relatively stronger than that to the left and right side of divacancy D. Therefore, the ability of phonons to penetrate D has a strong anisotropy, that is, the ability to penetrate through D in the vertical direction is much greater than the horizontal direction. In the 15-ZGNR, the thermal current is horizontal, so the phonon penetration is small and the thermal conductance is low. As a result of Figure 2, the thermal conductance of D is the smallest among the five structures, which indicates that the possibility of phonons penetrate D is smaller than both A and C. At the same time, comparing D with E, it is found that although the cross section of E is larger than D, the phonon has a strong ability to pass E along the strong strength part (marked by the dashed line in Figure 4a) to the upper right, and then finally the thermal conductance of E is larger than D.*

**Example** **2.** *Describe and explain the thermal conductance data when divacancies are located at the center of the 26-AGNR. Comparing among A, B and C, from the view point of only considering the cross section, the scattering possibility is ranked as A > B > C, then the sort of thermal conduction is A < B < C, which is consistent with Figure 2. This also implies that the difference between the ability of the phonon to penetrate the three defects in the vertical direction is not significant, otherwise it will interfere with the ordering of thermal conductance. Comparing D and E, although the cross section of D is larger, the ability of phonons to penetrate D vertically is much stronger than that of oblique penetration of E, so that the thermal conductance of D is larger than E.*

**Example** **3.** *Explain why the thermal conductance of the GNR with a divacancy in the center is lower than that at the edge. Especially for type A divacancy, the thermal conductance of A in the center of the 26-AGNR is the lowest one of all structures; while at the edge, it is the second highest. The defect structure and direction are the same whether at the edge or in the center, so the probability of phonons penetrating should not be much different. The difference is that, as showed in Figure 4d, the 585 defect at the boundary has only three additional hexagons. The cross section are much smaller than the 585 defect in the center. Smaller cross section, less phonons will collide with it. Moreover, considering the comparable probability of penetration, fewer phonons will be scattered, and there will be larger thermal conductance.*

### 3.2. Randomly Distributed Multiple Divacancies

Based on the results of one single divacancy, the influence of multiple randomly distributed divacancies in GNR on its thermal transport properties is also investigated. We use the cascade model to study the thermal transport properties of GNR with multiple randomly distributed divacancies, which has been successfully applied to compute the effects of multiple independent scatterers [64,79,80,81].

When there are *N* scatterers in the system (here is *N* divacancy defects), the transmission spectrum given by the cascade model is [64,79]

(3) $$\frac{1}{T_{N}}=\frac{N}{T_{i}}-\frac{N-1}{T_{0}},$$

where $T_{0}$ is the phonon transmission when there is no scatterer, $T_{i}$ is the transmission spectrum when there is only one defect scatterer, which is calculated by the Nonequilibrium Green’s function method.

**Figure 5:**
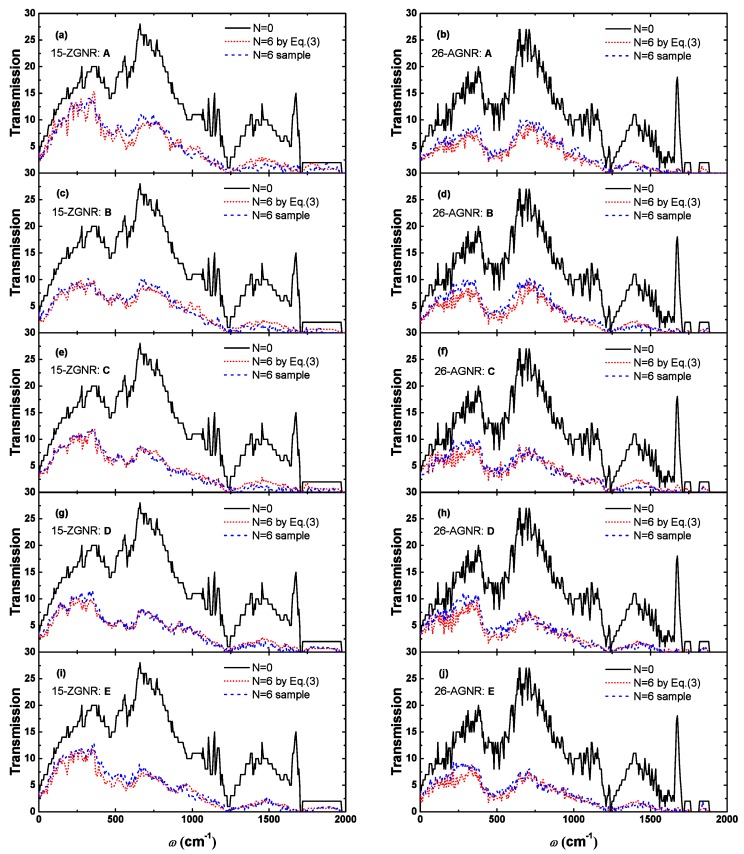
The phonon transmission of graphene nanoribbons with 6 randomly distributed divacancies. The left and right sides are transmission of 15-ZGNR and 26-AGNR, respectively. From top to bottom, there are transmission containing six divacancy defects of type A, B, C, D and E, respectively. The red dotted line in the figure is the transmission of the six defects calculated using the Equation (3); the blue dashed line is the averaged transmission of 5 randomly distributed samples directly calculated by the nonequilibrium Green’s function (NEGF).

The transmission calculated by the cascade model is showing in Figure 5. In order to check this approximate model, we directly calculated transmission of randomly distributed samples with the same number of defects by NEGF method. Among them, five randomly distributed samples were calculated for each type of divacancy, and then the average value is utilized to compare with the cascade model, as showed in Figure 5. Obviously, the phonon transmission obtained by the two methods are only slightly different, which indicate that the cascade model is suitable for describing the effects of multiple randomly distributed divacancies.

In addition, we find that multiple random divacancies not only have a great effect on the scattering of high-frequency phonons, but also lead to a significant decrease in the transmission spectra of low-frequency phonons, thereby the thermal conductance will clearly decrease. In the direct calculations of randomly distributed samples, the thermal conductances of 15-ZGNR and 26-AGNR with six randomly distributed divacancies are greatly reduced: in the 15-ZGNR, the decline ranges from 45% (model A) to 56% (model D); in the 26-AGNR, the decline ranges from 53% (model D) to 58% (model E).

**Figure 6:**
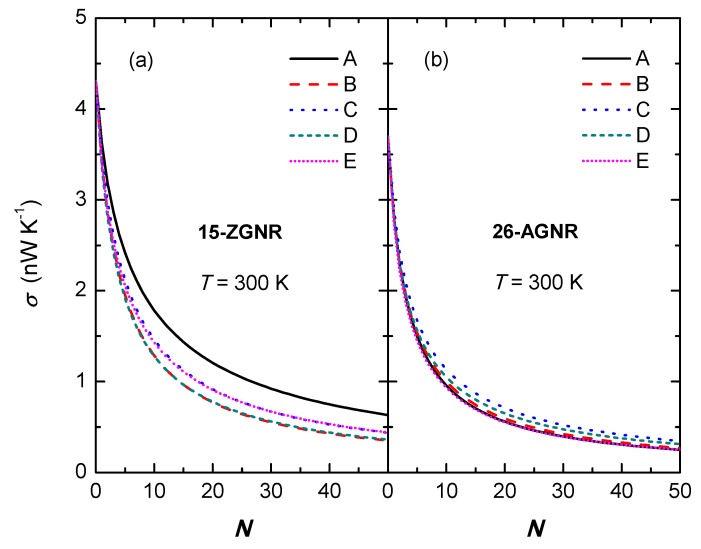
The thermal conductance versus the number of divacancy defects *N* at 300 K, on (**a**) 15-ZGNR and (**b**) 26-AGNR, respectively.

Figure 6 indicates the relationship between thermal conductance and the number of divacancy *N* at 300 K. We find that in the case of low amount defects, the thermal conductance declines rapidly with the increase of divacancies; while in the case of high amount defects, the thermal conductance decreases slowly with the increase of divacancies, and finally tends to be saturated. This saturation behaviour is a common phenomenon and it has also appeared in other theoretical and experimental results [78,82,83,84]. For the 26-AGNR, with the change in the amount of divacancies for different types of divacancy, the numerical differences of their thermal conductances are not significant; but for the 15-ZGNR, the thermal conductances of GNRs with model-A defects are obviously greater than those of other configurations. Moreover, the thermal conductance of the 15-ZGNR is greater than that of the 26-AGNR regardless of the amount of divacancies, which indicates that the anisotropy of the thermal conductance is still present.

Some structures may have more than one type of divacancies at the same time. The combined cases of different divacancies can not be described directly by the cascade model. However, we can still expect that the conductance of combined configurations should mostly be located between those lines in Figure 6.

### 3.3. Extended Line Of Divacancies

**Figure 7:**
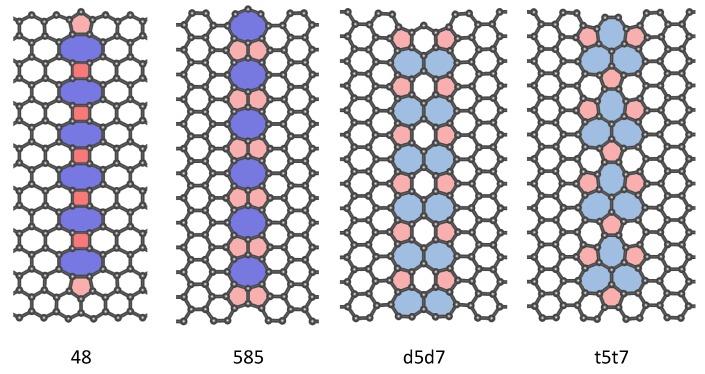
The structure diagrams of four kinds of divacancy extended line of defects (ELD), which are calculated herein. The heat transport direction is perpendicular to the ELD chain. Notices that, this figure just presents the part near ELD, which is a short part of center transport region.

**Figure 8:**
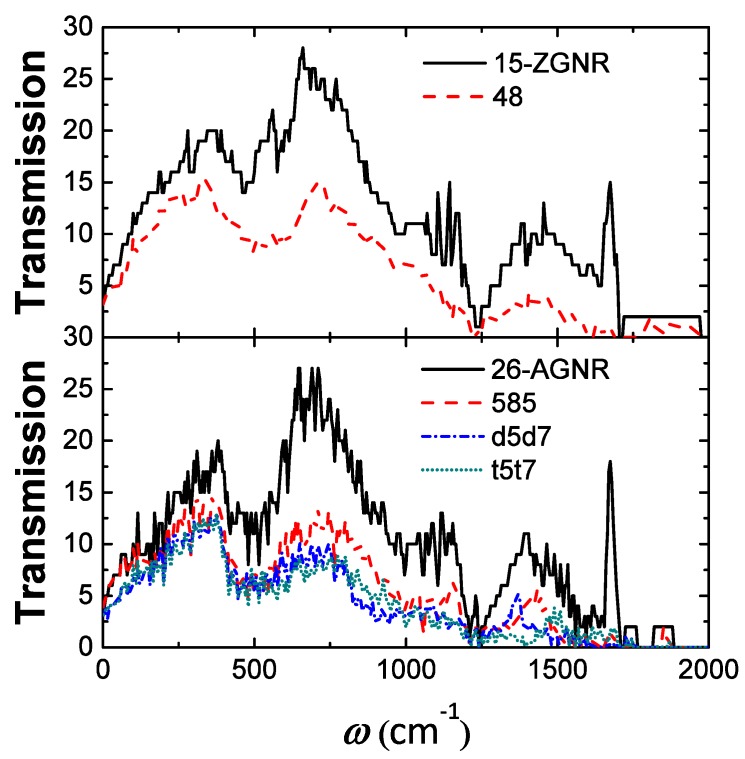
Transmission of graphene nanoribbon containing a single divacancy ELD.

Multiple divacancies may also form an extended line of defects (ELD) as shown in Figure 7. Not only are the 585, d5d7 (5577) and t5t7 (555777) ELD embedded in AGNRs investigated, but also the 48 ELD embedded in ZGNRs. The thermal conductances of GNRs with the four kinds of ELD at the temperature of 300 K are summarised in Table 2 respectively. We find that when the ELD is perpendicular to the transport direction, it will greatly reduce the thermal conductance of the GNR, resulting in a decrease of about 33% to 47% of the total, the results are in accordance with the previous work [55], where the largest decline are caused by the d5d7 and t5t7 ELDs. From the variation of transmission spectra (Figure 8), it can be known that the 48, D5d7 and t5t7 ELD not only have an influence on the scattering of high-frequency phonons, but also have a strong influence on the scattering of low-frequency phonons. The scattering of the phonon ($\omega <300\;{\mathrm{cm}}^{-1}$) caused by the 585 ELD is not significant, thus the decrease of thermal conductance is relatively small.

As shown in Table 2, we also compare the thermal conductance of the GNR with ELD and that with the same number of randomly distributed divacancies. It is found that the thermal conductances of GNRs with multiple t5t7 divacancies are almost the same, whether the t5t7 divacancies are randomly distributed or formed an ELD. However, for the 585 divacancies, the influence of the ELD on the thermal conductance is less, i.e., the thermal conductance decreases by 33% when the ELD was formed, and the decline of the thermal conductance ranges from 54% to 57% when the divacancies are randomly distributed. Formation of the 585 ELD results in a further reconstruction of the system, while the interaction between the single divacancies is improved. Analyzing from the phonon transmission, the 6 randomly distributed 585 divacancies will scatter both low-frequency and high-frequency phonons. However, there is no scattering in the low-frequency phonons ($\omega <300\;\mathrm{cm}{}^{-1}$) when the ELD was formed, which results in a higher thermal conductance compared with that of the same number of randomly distributed 585 divacancies. The reconstruction and interaction between the single divacancies is weak when the t5t7 ELD is formed. Thus the thermal transport properties are similar to those when the divacancies are randomly distributed.

## 4. Conclusions

The influence of divacancies with different configurations and amounts on the thermal transport properties of armchair and zigzag GNRs is investigated by the nonequilibrium Green’s function method. For a single divacancy, the position, direction and structure are taken into consideration. We find that the thermal conductance of GNRs can be greatly reduced by divacancy defects, the influence of divacancies on the thermal transport properties of AGNRs is greater than that of ZGNRs, and the scattering of phonons when a divacancy is in the center region of GNR is stronger than that at the ribbon edge. The boundary effect is explained by the collective effect of impurity scattering and boundary scattering, and the different effects of configuration or position are explained by the combined method of the cross-sectional size and the internal structure. Multiple randomly distributed divacancies have a significant impact on the scattering of high-frequency phonons as well as low-frequency phonons. The same trend was seen among the thermal conductances of ZGNRs and AGNRs varying with the change in amount of divacancies with different configurations. Specifically, in the case of a low amount, the thermal conductance declines rapidly with the increase of divacancies, while in the case of a high amount, the thermal conductance decreases slowly with the increase of divacancies. We also investigated the case of multiple divacancies forming an ELD, it is noted that the 48, d5d7 and t5t7 ELD have an influence on the scattering of both low-frequency and high-frequency phonons, while the 585 ELD has no influence on the scattering of low-frequency phonons ($\omega <300\;{\mathrm{cm}}^{-1}$). Finally, we compared the thermal conductance of GNR with multiple random divacancies with the ELDs which are formed with the same number of corresponding divacancies. It is found that, whether the t5t7 divacancies are randomly distributed or formed an ELD, the thermal conductances of GNRS are almost the same. However, for the 585 divacancies, the thermal conductance of GNR is higher when the 585 divacancies formed an ELD than when they are randomly distributed.

## Figures and Tables

**Table 1:** Thermal conductance σ of graphene nanoribbon containing a single divacancy in center transport region at 300 K. (Unit: nW $K^{-1}$).

		A	B	C	D	E
**15ZGNR** ${}^{1}$	**Center**	3.630	3.320	3.432	3.298	3.367
	**Edge**	3.608	3.450	3.531	3.412	3.435
**26AGNR** ${}^{2}$	**Center**	2.673	2.773	2.804	2.746	2.675
	**Edge**	2.937	2.845	2.983	2.818	2.790

${}^{1}$ conductance of pristine 15ZGNR $\sigma_{0}$ = 4.294 nW $K^{-1}$. ${}^{2}$ conductance of pristine 26AGNR $\sigma_{0}$ = 3.686 nW $K^{-1}$.

**Table 2:** Thermal conductance σ of graphene nanoribbon containing a single divacancy ELD at 300 K. (Unit: nW $K^{-1}$).

	Zigzag	Armchair		
	48	585	d5d7	t5t7
σ	2.797	2.466	2.016	1.958
$\sigma /\sigma_{0}$	65.1%	66.9%	54.7%	53.1%
$\sigma_{N}$ ${}^{1}$	–	1.588 ($N_{A}$ = 6) 1.700 ($N_{B}$ = 6)	–	1.921 ($N_{C}$ = 4)

${}^{1}$ Randomly distributed multiple defects with the same amount of divacancies.

## Abbreviations

The following abbreviations are used in this manuscript:

GNR	Graphene nanoribbon
NEGF	Nonequilibrium Green’s function
ELD	Extended line defect
*N*-ZGNR	Graphene nanoribbon with *N* zigzag carbon chains in width direction
*N*-AGNR	Graphene nanoribbon with *N* dimer carbon chains in width direction
